# Spatiotemporal variability in population demography and morphology of the habitat-forming macroalga *Saccorhiza polyschides* in the Western English Channel

**DOI:** 10.1093/aob/mcad181

**Published:** 2023-11-14

**Authors:** Nora Salland, Catherine Wilding, Antony Jensen, Dan A Smale

**Affiliations:** The Marine Biological Association of the United Kingdom, The Laboratory, Citadel Hill, Plymouth PL1 2PB, UK; School of Ocean and Earth Science, University of Southampton, European Way, Southampton SO14 3ZH, UK; The Marine Biological Association of the United Kingdom, The Laboratory, Citadel Hill, Plymouth PL1 2PB, UK; School of Ocean and Earth Science, University of Southampton, European Way, Southampton SO14 3ZH, UK; The Marine Biological Association of the United Kingdom, The Laboratory, Citadel Hill, Plymouth PL1 2PB, UK

**Keywords:** *Saccorhiza polyschides*, Furbelows, kelp, marine forests, seaweeds, macroalgae, foundation species, habitat-former, temperate rocky reefs, seasonality, wave exposure, ocean warming

## Abstract

**Background and Aims:**

Large brown macroalgae serve as foundation organisms along temperate and polar coastlines, providing a range of ecosystem services. *Saccorhiza polyschides* is a warm-temperate kelp-like species found in the northeast Atlantic, which is suggested to have proliferated in recent decades across the southern UK, possibly in response to increasing temperatures, physical disturbance and reduced competition. However, little is known about *S. polyschides* with regard to ecological functioning and population dynamics across its geographical range. Here we examined the population demography of *S. polyschides* populations in southwest UK, located within the species’ range centre, to address a regional knowledge gap and to provide a baseline against which to detect future changes.

**Methods:**

Intertidal surveys were conducted during spring low tides at three sites along a gradient of wave exposure in Plymouth Sound (Western English Channel) over a period of 15 months. Density, cover, age, biomass and morphology of *S. polyschides* were quantified. Additionally, less frequent sampling of shallow subtidal reefs was conducted to compare intertidal and subtidal populations.

**Key Results:**

We recorded pronounced seasonality, with fairly consistent demographic patterns across sites and depths. By late summer, *S. polyschides* was a dominant habitat-former on both intertidal and subtidal reefs, with maximum standing stock exceeding 13 000 g wet weight m^−2^.

**Conclusions:**

*Saccorhiza polyschides* is a conspicuous and abundant member of rocky reef assemblages in the region, providing complex and abundant biogenic habitat for associated organisms and high rates of primary productivity. However, its short-lived pseudo-annual life strategy is in stark contrast to dominant long-lived perennial laminarian kelps. As such, any replacement or reconfiguration of habitat-forming macroalgae due to ocean warming will probably have implications for local biodiversity and community composition. More broadly, our study demonstrates the importance of high-resolution cross-habitat surveys to generate robust baselines of kelp population demography, against which the ecological impacts of climate change and other stressors can be reliably detected.

## INTRODUCTION

Kelps – large brown macroalgae – are widely distributed across the world’s temperate and polar coastlines (Jayathilake and Costello, 2021), where they form vital ‘marine forest’ habitats (Duarte *et al.*, 2022). These forests typically exhibit high levels of biodiversity and primary productivity (Steneck *et al.*, 2002; Teagle *et al.*, 2017; King *et al.*, 2021; Smale *et al.*, 2021; Pessarrodona *et al.*, 2022*a*; United Nations (UN) Environment Programme, 2023), and provide a range of ecosystem services with direct and indirect socioeconomic value (Smale *et al.*, 2013; Eger *et al.*, 2023). While the ecological structure and function of kelp species vary to some extent across regions and habitats, they generally serve as foundation organisms within coastal ecosystems (Lüning, 1985; Steneck *et al.*, 2002; Smale *et al.*, 2013).

Kelp species dominate shallow rocky habitats along the extensive coastline of the northeast Atlantic (Smale *et al.*, 2013). Whereas some kelp species have been intensively studied (e.g. *Laminaria hyperborea*, *L. digitata*, *Saccharina latissima*) (e.g. Kain, 1979; Bartsch *et al.*, 2008; Diehl *et al.*, 2023 and references therein), information on the distribution and demography for other species (e.g. *Saccorhiza polyschides*, *Laminaria ochroleuca*, *Alaria esculenta*) remains more spatially limited or lacking for many areas. Furthermore, species and populations inhabiting intertidal and/or wave-sheltered environments are generally better studied than those occupying subtidal and/or wave-exposed habitats (Smale *et al.*, 2013; Araújo *et al.*, 2016), due to their restricted accessibility.

### Impacts of climate change on kelp forests

Marine forests are currently threatened by a range of human-mediated stressors, including climate change factors (e.g. ocean warming and marine heatwaves, ocean acidification, increased storminess, rising sea levels), the spread of invasive species, fishing impacts (e.g. mechanical damage and sedimentation from trawling, and trophic cascades) and decreased coastal water quality (, Harley *et al.*, 2006; Filbee-Dexter *et al.*, 2020; Norderhaug *et al.*, 2020; Smith *et al.*, 2021*b*; IPCC, 2022). Ocean warming, in particular, is emerging as a pervasive driver of ecosystem change, causing species range shifts, local and regional extirpations, species replacements, altered ecological interactions, changes in population demography and community composition, and even reconfigurations of entire ecosystems (Hawkins *et al.*, 2009; Merzouk and Johnson, 2011; Harley *et al.*, 2012; Koch *et al.*, 2013; Araújo *et al.*, 2016; Assis *et al.*, 2018; Smale, 2020). In some regions, the ‘tropicalization’ of former kelp beds, the rise of turf algae and shifts to barren grounds caused by increased herbivory (from sea urchins, for example) have been observed and attributed to ocean warming and related environmental changes (Vergés *et al.*, 2014; Pessarrodona *et al.*, 2021, 2022*b*). Ecological responses of marine forests to anthropogenic climate change can also have substantial socioeconomic impacts (Smith *et al.*, 2021*a* and references therein).

### Species range shifts in the NE Atlantic related to ocean warming, and long-term monitoring efforts

In the northeast Atlantic, recent ocean warming trends have affected several forest-forming macroalgal species across multiple regions, most often manifesting as species range shifts or changes in population demography (Assis *et al.*, 2018; Smale, 2020). For example, populations of *Laminaria hyperborea*, *L. ochroleuca* and *Saccorhiza polyschides* have declined towards their southern (trailing) range edges and, conversely, proliferated and expanded towards their northern (leading) edges (Müller *et al.*, 2009; Fernández, 2011; Smale *et al.*, 2013; Casado-Amezúa *et al.*, 2019). In addition, local extinctions of populations found towards species’ trailing range edges have also been observed (Fernández, 2011; Díez *et al.*, 2012; Casado-Amezúa *et al.*, 2019).

Along parts of the coastline of the UK and Ireland, warm-tolerant species such as *L. ochroleuca* and the non-native kelp *Undaria pinnatifida* have proliferated and expanded their distributions (Epstein and Smale, 2017*b*; Teagle and Smale, 2018; Schoenrock *et al.*, 2019), whereas some more northerly distributed cold-adapted species, such as *Alaria esculenta*, have undergone population declines and probable range contractions (Simkanin *et al.*, 2005; Birchenough and Bremner, 2010).

It is likely, however, that other impacts of ocean warming on marine forests have gone undetected and under-reported, due to a lack of baseline data and monitoring efforts in many areas. That said, in some well-studied regions, long-term monitoring of kelp forests using a range of approaches, such as snorkelling and diving, towed video, remotely operated vehicles, satellite-derived imagery, and citizen/community science initiatives, has reliably quantified temporal trends (Thompson, 2021; Reshitnyk *et al.*, 2023). Even in regions or countries with a strong track record of marine monitoring, however, tropical ecosystems have received far more resources and attention than their temperature counterparts, which are typically dominated by kelp forests (e.g. Australia, see Bennett *et al.*, 2015). Historically, the NE Atlantic is a relatively data-poor region of macroalgae research, particularly concerning sustained observation and monitoring (Smale *et al.*, 2013) when compared to well-studied ecosystems such as the Giant Kelp forests (*Macrocystis pyrifera*) along the Californian coast (Schiel and Foster, 2015).

A deeper understanding of spatiotemporal variability patterns in kelp population demographics persisting under a range of environmental conditions and stressors is needed to monitor and predict responses to environmental changes. Given that kelp populations exhibit high levels of intra- and inter-annual variability due to, for example, temporal growth strategies and winter storm disturbances (Kain, 1979; Seymour *et al.*, 1989; Smale and Vance, 2015), it is imperative that any efforts to address kelp demography capture seasonal changes. Similarly, kelp species and populations are highly variable across spatial gradients, as morphology, density and standing stock are strongly influenced by key abiotic and biotic variables (e.g. wave exposure, light availability, grazing pressure). As such, spatial variability should be considered in any baseline survey. While sustained observations through long-term monitoring represent the optimal approach for detecting climate change impacts, monitoring programmes are generally costly and logistically challenging and are lacking for most kelp species, habitats and regions. Citizen or community science initiatives represent a useful low-cost tool to collect long-term data while engaging the local stakeholders in marine research and drawing attention to the health of marine species, habitats and ecosystems (Sandahl and Tøttrup, 2020; Fraisl *et al.*, 2022). Community science projects that involve kelp forest observations are, for example, currently undertaken by Reef Check in California, Reef Life Survey in temperate Australia and Seasearch along parts of the British coast (Bull *et al.*, 2013; Freiwald *et al.*, 2021; Lucrezi, 2021; Edgar *et al.*, 2023). However, in the absence of long-term monitoring or well-established community science initiatives, appropriately designed focused surveys that target key or indicator species can fill knowledge gaps, provide a baseline against which to detect changes and inform about the general condition of the ecosystem.

We focus on one kelp species, *Saccorhiza polyschides*, which is expected to proliferate under ongoing ocean warming and other environmental changes, in a biogeographical transition zone in the Western English Channel (Dinter, 2001; Dauvin, 2019). We examine spatiotemporal variability in demography to generate a robust baseline on range-centre populations that can be used to predict, detect and test future changes of a habitat-forming kelp species under climate change.

### Saccorhiza polyschides in the NE Atlantic

*Saccorhiza polyschides* (Lightfoot) Batters 1902, commonly known as ‘Furbelows’, is a large canopy-forming brown macroalga belonging to the order Tilopteridales. Although not a ‘true kelp’ in the traditional taxonomic definition (i.e. belonging to the order Laminariales), it serves a similar ecological function as other marine forest formers and is commonly included within kelp assemblages (Smale *et al.*, 2013). *Saccorhiza polyschides* sporophytes become fertile and reproduce in late summer/early autumn, with new recruits appearing in the following spring. Recruits exhibit rapid growth through summer and autumn to mature sporophytes before the onset of senescence (Norton and Burrows, 1969). As the overall lifespan of the macroscopic sporophyte does not exceed 12–18 months (Sauvageau, 1915; Norton and Burrows, 1969), *S. polyschides* is a so-called ‘pseudo-annual’ species, whereas most ‘true’ kelp species in the NE Atlantic are longer-lived, multi-year perennials (i.e. *Laminaria* sp.). Due to its fast growth and high productivity within one season, *S. polyschides* has been described as an opportunistic pioneer species (Norton and Burrows, 1969; Kain and Jones, 1975; Assis *et al.*, 2013; Smale *et al.*, 2013; Pereira, 2014; Fernández *et al.*, 2022).

As with other kelp species, sporophytes of *S. polyschides* comprise three main morphological components: a digitated blade (i.e. lamina), stipe and holdfast. Unlike true kelps, however, *S. polyschides* exhibits a distinctive hollow bulbous holdfast with many haptera, converging into a flattened stipe, which is twisted at its base just above the holdfast (see Barber, 1889). Kelp sporophytes, and especially their holdfasts, provide an important habitat for faunal assemblages by offering shelter, nursery grounds and increased food supply for invertebrates (McKenzie and Moore, 1981; Thiel and Vásquez, 2000; Feehan *et al.*, 2014; Teagle *et al.*, 2017, 2018) and coastal fishes (McKenzie and Moore, 1981; Gordon, 1983; Salland and Smale, 2021; Christie *et al.*, 2022). The large bulbous holdfast of *S. polyschides* offers plentiful living space for fauna (Norton, 1971), and it can persist for much of the year even following the senescence of the rest of the sporophyte, representing important habitat for over-wintering assemblages (Salland and Smale, 2021).

*Saccorhiza polyschides* extends from the low intertidal zone into subtidal habitats to depths of 25 m or more (Arnold *et al.*, 2016; Smale and Moore, 2017; D. A. Smale, unpubl. data) and is distributed in the NE Atlantic from the Mediterranean and Morocco polewards to Norway and the Faroes (Norton, 1977; Lüning, 1985; Smale *et al.*, 2013). In warmer, more southerly regions within its latitudinal distribution, *S. polyschides* is often the dominant habitat-former and exhibits high population densities (Fernández and Niell, 1982; Fernández, 2011), while in cooler, more northerly regions it tends to be outcompeted by *Laminaria* species and is generally found in mixed stands or restricted to disturbed or marginal habitats (Hawkins and Harkin, 1985; Smale and Moore, 2017). Limited evidence suggests that this species has increased in abundance and spatial extent along the southern coastline of the UK in recent decades (Birchenough and Bremner, 2010; Smale *et al.*, 2013). This is perhaps in response to rising temperatures becoming more favourable for its performance, as well as increased disturbance to *Laminaria* forests reducing competition and other environmental changes (Kain and Jones, 1975). It has therefore been described as a potential ‘winner’ of ocean warming (Smale *et al.*, 2013), particularly for populations found towards the range centre or leading range edge, such as those in UK waters. However, only limited data on local *S. polyschides* populations in SW England are available to confirm this assumption and the empirical evidence base remains limited. Notwithstanding some early natural history work on its morphology, demography and ecological role within the ecosystem (Barber, 1889; Burrows, 1958; Norton, 1969; Norton and Burrows, 1969), the only recent study focused on *S. polyschides* in the UK and across the wider central area of its latitudinal distribution examined spatial variability in sporophyte density and morphology from a single sampling event (Salland and Smale, 2021).

### Aim of baseline study on S. polyschides populations in a biogeographical transition zone

Given the role of *S. polyschides* as a habitat-forming foundation species and the rapid growth rates observed for sporophytes through spring and summer, this species may be important for local biodiversity and primary productivity, yet robust information on population dynamics within a biogeographical transition zone (i.e. the Western English Channel, see Dinter, 2001; Southward *et al.*, 2005; Cox *et al.*, 2016; Dauvin, 2019) and its range-centre is lacking. Without this knowledge, it is not possible to detect nor assess the wider ecological consequences of future changes. We conducted surveys at three intertidal rocky shores over 15 months, as well as a less frequent survey of three subtidal reefs, to quantify the structure of *S. polyschides* populations within a region of probable climate-driven expansion, to address this pressing knowledge gap.

## MATERIALS AND METHODS

### Survey design

**Fig. 1.**
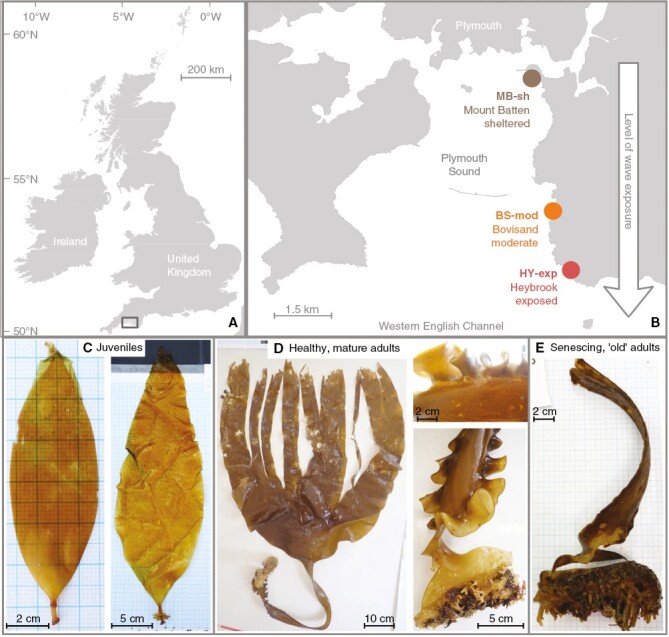
Survey location and morphological differences between sporophyte age classes of *S. polyschides.* (A) Map indicating region of study area in southwest England (grey box) and (B) location of three sites along a wave exposure gradient in Plymouth Sound. (C–E) Representative examples of the three age/size classes of *S. polyschides*: (C) juvenile recruits with a total length < 30 cm and incomplete development of bulbous holdfast; (D) healthy adult ‘plants’ with total length > 30 cm, complete development of closed, bulbous holdfast (bottom right), possible presence of fertile sporophyll tissue (top right) and some limited tissue loss at the distal tips of blades caused by grazing and wave action; (E) senescing, ‘old’ adults exhibiting partial or complete decay of blade, stipe and/or holdfast.

Surveys of intertidal *S. polyschides* populations were conducted every month during periods of spring low tides at a tidal height of +0.5–0.8 m (relative to chart datum) at three rocky shore sites in and around Plymouth Sound (50°N), southwest UK. A mean tidal range of 4.7 m influences Plymouth Sound and sea temperature typically ranges between 8 and 18 °C throughout the year (Pessarrodona *et al.*, 2018*b*). The three survey sites were situated along a gradient of wave exposure (Supplementary Data S1) from semi-sheltered Mount Batten (MB-sh; protected by the coastline of the Sound and a man-made breakwater structure) to moderately sheltered/exposed Bovisand (BS-mod; open towards the Western English Channel, partly sheltered from prevailing swell by an adjacent headland) to fully exposed Heybrook Bay (HY-exp; open to the Western English Channel with minimal protection) (Fig. 1) (Salland and Smale, 2021). All sites were deemed representative of the wider region (i.e. shallow rocky reef with silt/sandy patches and deeper gullies; see Supplementary Data S1) and were without obvious local anthropogenic impacts (i.e. pollution, trawling).

Surveys commenced in February 2020 (survey month 1) and continued until April 2021 (survey month 15). Due to adverse weather conditions, surveys were not feasible in January 2021 (survey month 12) and therefore data were not collected in this month. Additionally, *S. polyschides* populations on adjacent shallow subtidal reefs (2–4 m below chart datum) at all three sites were surveyed by SCUBA diving on three occasions (June, August and October 2020; survey months 5, 7 and 9, respectively) to examine differences in population demography and morphology with increasing water depth.

### Density and cover

During each survey, ten 1-m^2^ quadrats were haphazardly placed in areas of appropriate habitat (stratified for emergent rocky substrate rather than rock pools or sandy patches) within the appropriate tidal height, positioned at least 2 m apart from one another. The density (number of individuals) and cover (visually estimated as a percentage) of *S. polyschides* sporophytes of each age class were recorded *in situ*. Age classes were defined as either juveniles, healthy adults, or senescent adults based on a modified classification scheme from Norton and Burrows (1969) (detailed further in Fig. 1 and Supplementary Data S2). The cover of ‘other kelps’ (i.e. *L. hyperborea*, *L. ochroleuca*, *L. digitata*, *Saccharina latissima* and *U. pinnatifida*) was also recorded (from May 2020 onwards; visual estimation). Due to the three-dimensional multi-layered structure within the water column in a marine forest, estimates of total percentage cover often exceeded 100 % since different species and size classes overlap within the quadrat (Smale and Moore, 2017).

### Sporophyte biomass and morphology

During each survey, ten representative sporophytes per site and month were randomly collected by carefully removing each one from the substratum and transferring it into a cotton bag. These samples were taken from a separate part of the shore to the density quadrats. On return to the laboratory, individuals were assigned to one of the three age classes, photographed, measured and weighed. Measurements of length and fresh weight biomass (blotted tissue dry) were obtained separately for the different sporophyte components (i.e. holdfast, stipe and blade), while the weight of the sporophyll (reproductive frills) and mature sorus tissue was obtained where present. Sorus tissue forms ‘a palisade-like cell layer at the surface of the thallus’ (Norton and Burrows, 1969), which permits visible detection of the mature tissue.

### Estimation of standing stock and biomass accumulation

To estimate the standing stock of *S. polyschides*, mean density values were multiplied by mean sporophyte biomass for each month and site, to yield g wet weight m^−2^ (g WW m^−2^). Maximal biomass accumulation (maximal standing stock), the gain of biomass from a juvenile recruit to a fully grown mature sporophyte, was used to estimate total biomass accumulation during a growing season, as a proxy for primary productivity.

### Statistical analysis

Between-site and between-month variation in response variables was examined with univariate permutational analysis of variance (PERMANOVA), conducted with R version 4.0.0 (R Core Team, 2020), R Studio and PRIMER 7.0.21 (Anderson *et al.*, 2008; Clarke *et al.*, 2014). If not stated otherwise, results are given as monthly means with standard error (± s.e.) per site and depth. Data (untransformed) were used to construct similarity matrices based on Euclidean distances (dummy value of ‘1’). Intertidal (three sites, 14 months) and subtidal (three sites, three months) datasets were analysed separately with ‘site’ and ‘month’ as fixed factors (9999 permutations under a reduced model with Monte Carlo correction). Pairwise post-hoc tests were conducted where significant effects were detected.

## RESULTS

### Density and cover

**Fig. 2.**
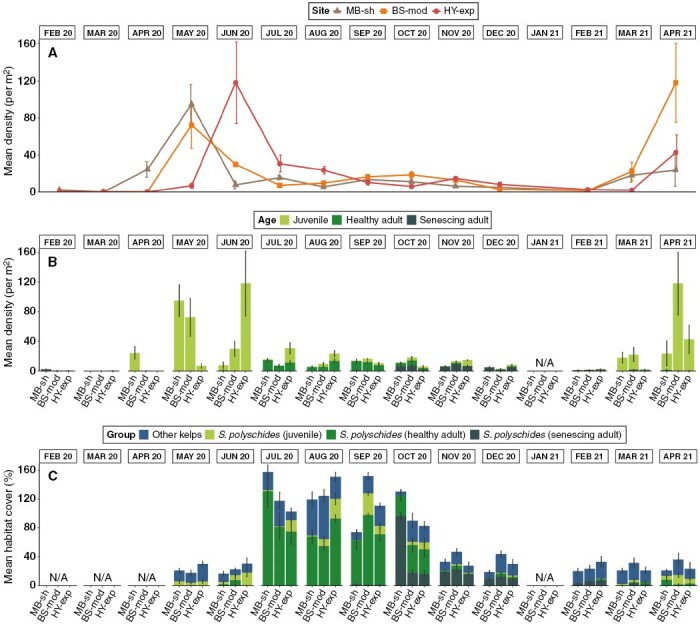
Spatiotemporal variability in density and cover of *S. polyschides* populations in intertidal habitats at three sites (with increasing levels of wave exposure from left, MB-sh, to right, HY-exp) in Plymouth Sound over 15 months. (A) Mean total density (± s.e.) and (B) mean density of each age class at each site and month during the intertidal survey. (C) Mean habitat percentage cover (± s.e.) of *S. polyschides* (differentiated between three age classes) and other kelps (*Laminaria* spp., *Saccharina latissima*, *U. pinnatifida*) recorded at each site and month.

The density of *S. polyschides* sporophytes and the composition of age classes across the year in the intertidal zone exhibited high variability between months, following a seasonal pattern of recruitment and growth (Fig. 2A, B). Mean total density ranged from 0 ± 0 to 118 ± 44 individuals m^−2^.

Juvenile sporophytes dominated in spring and early summer (March/April until June), with mean density increasing more than 100-fold over 2–3 months. Maximum mean density of juveniles was observed at the more wave-exposed sites HY-exp in June 2020 and at BS-mod in April 2021, at 118.1 ± 44 and 118 ± 43 individuals m^−2^, respectively. Following rapid growth of recruits, populations were dominated by an ‘older’, mature adult sporophyte cohort in summer and autumn (July–October), with a maximum mean density of 14.4 ± 2.4 individuals m^−2^ recorded at MB-sh in July 2020. In the winter months (November–February/March), populations comprised senescing ‘old’ adults. The maximum mean density of senescing sporophytes (8.8 ± 1.7 individuals m^−2^) was recorded at BS-mod in November 2020. We observed a smaller cohort of late recruits, appearing in late summer, which did not become fertile until the following year. We also observed an overlap between decaying holdfasts, late recruits and newly observed recruits in late winter 2020 and early spring 2021.

Kelps (i.e. *S. polyschides* and Laminariales) dominated the intertidal habitat between July and October, covering more than half of the surveyed shore (Fig. 2C). The cover of *S. polyschides* increased markedly through summer into autumn, becoming the dominant space occupier between July and October (ranging from 132.6 ± 7.9 % at MB-sh in July to 60 ± 11.4 % at HY-exp in October).

For all response variables, univariate PERMANOVA detected a highly significant effect of the ‘site × month’ interaction term, as well as the main effect of ‘month’ (Table 1). However, there were no significant differences between the term ‘site’ in density and cover. Post-hoc comparisons within levels of the interaction term showed that the magnitude of differences between ‘month’ were not always consistent between sites and therefore along the wave exposure gradient (Supplementary Data S3).

**Table 1.** Results of a univariate PERMANOVA to test for differences in intertidal kelp density, cover and biometric measurements between sites and sampling months of intertidal *S. polyschides* populations. PERMANOVAs (9999 permutations) are based on Euclidean distances with a dummy value of 1, under a reduced model with Monte Carlo (MC) correction. ‘Site’ and ‘month’ as fixed factors. Significant values are indicated in bold (*P* ≤ 0.05). Post-hoc pairwise test followed PERMANOVAs (Supplementary Data S3).

Response variable	Site			Month			Site × month			
Intertidally	d.f.	*F*	*P* (MC)	d.f.	*F*	*P* (MC)	d.f.	F	*P* (MC)	Res d.f.
**Density of *S. polyschides* per m** ^**2**^										
*S. polyschides* (sum)	2	0.48	*0.6223*	13	8.9854	***0.0001***	26	4.9851	***0.0001***	378
**Cover of *S. polyschides* per m** ^**2**^										
*S. polyschides* (sum)	2	0.152	*0.8539*	11	68.28	***0.0001***	22	5.5141	***0.0001***	324
**Biometrics per *S. polyschides* individual**										
Total biomass/WW	2	8.6256	***0.0004***	13	51.211	***0.0001***	26	2.2436	***0.0005***	378
Total length	2	13.501	***0.0001***	13	134.7	***0.0001***	26	2.4561	***0.0001***	378
Sorus biomass/WW	2	21.049	***0.0001***	13	22.738	***0.0001***	26	8.3645	***0.0001***	378
**Standing stock**										
g WW m^−2^	2	2.197	*0.1116*	13	15.872	***0.0001***	26	3.275	***0.0001***	378

**Fig. 3.**
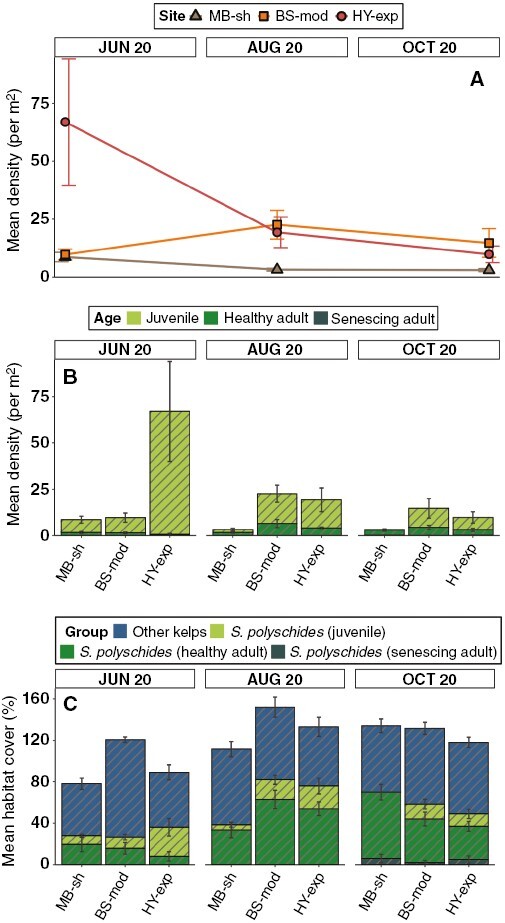
Spatiotemporal variability in density and cover of *S. polyschides* populations in subtidal habitats. (A) Mean total density (± s.e.), and (B) mean density of age classes at each site (along a gradient of wave exposure) and month. (C) Mean habitat percentage cover (± s.e.) of *S. polyschides* (differentiated between three age classes) and other kelps (*Laminaria* spp., *Saccharina latissima*, *U. pinnatifida*). Note that subtidal survey months are not consecutive.

For subtidal populations (sampled only in June, August and October), maximum mean density was observed in June (67 ± 27.4 individuals m^−2^ at HY-exp), dominated by juvenile recruits. This was followed by high adult density in August (22.6 ± 6.2 individuals m^−2^ at BS-mod), and a mixed stand of healthy and senescing adults in October (14.7 ± 6.2 individuals m^−2^ at BS-mod) (Fig. 3A, B). We recorded juvenile recruits at all subtidal sampling events with the exception of MB-sh in October. The coverage of *S. polyschides* contributed to more than half of all subtidal habitat-forming seaweeds at BS-mod and HY-exp in August, and at MB-sh and BS-mod in October (Fig. 3C). In August, *S. polyschides* was the most dominant kelp at all sites, but in June and October, the contribution of other kelps exceeded that of *S. polyschides*. Univariate PERMANOVA (Table 2) detected a significant ‘site × month’ interaction for both density and cover, but a main effect of ‘site’ was only detected for density and a main effect of ‘month’ was only detected for cover. Post-hoc comparisons within levels of the interaction term for density showed that the magnitude of differences between ‘month’ was not always consistent between sites (Supplementary Data S4), but for coverage, we could detect no differences between sites in June and October (Supplementary Data S4). In general, the density of *S. polyschides* in subtidal habitats was lower than that observed in intertidal habitats for corresponding months, but the cover of *S. polyschides* was broadly comparable.

**Table 2.** Results of univariate PERMANOVA for subtidal kelps in June, August and October 2020. PERMANOVAs (9999 permutations) are based on Euclidean distances with a dummy value of 1, under a reduced model with Monte Carlo (MC) correction. ‘Site’ and ‘month’ as fixed factors. Significant values are indicated in bold (*P* ≤ 0.05). Post-hoc pairwise test followed PERMANOVAs (Supplementary Data S4).

Response variable	Site			Month			Site × month			
subtidally	d.f.	*F*	*P* (MC)	d.f.	*F*	*P* (MC)	d.f.	*F*	*P* (MC)	Res d.f.
**Density of *S. polyschides* per m** ^**2**^										
*S. polyschides* (sum)	2	5.6284	***0.0047***	2	2.9474	*0.0568*	4	3.5225	***0.0114***	81
**Cover of *S. polyschides* per m** ^**2**^										
*S. polyschides* (sum)	2	1.0583	*0.3504*	2	12.785	***0.0001***	4	3.6592	***0.0078***	81
**Biometrics per *S. polyschides* individual**										
Total biomass/WW	2	0.3	*0.7434*	2	13.556	***0.0001***	4	4.7573	***0.0015***	81
Total length	2	5.2642	***0.0066***	2	7.2153	***0.001***	4	1.4334	*0.2223*	81
Sorus biomass/WW	2	0.2506	*0.78*	2	7.2847	***0.0012***	4	4.4964	***0.0027***	81
**Standing stock**										
g WW m^−2^	2	3.9109	***0.0231***	2	0.3802	*0.6829*	4	0.9631	*0.4316*	81

### Biomass and morphology

**Fig. 4.**
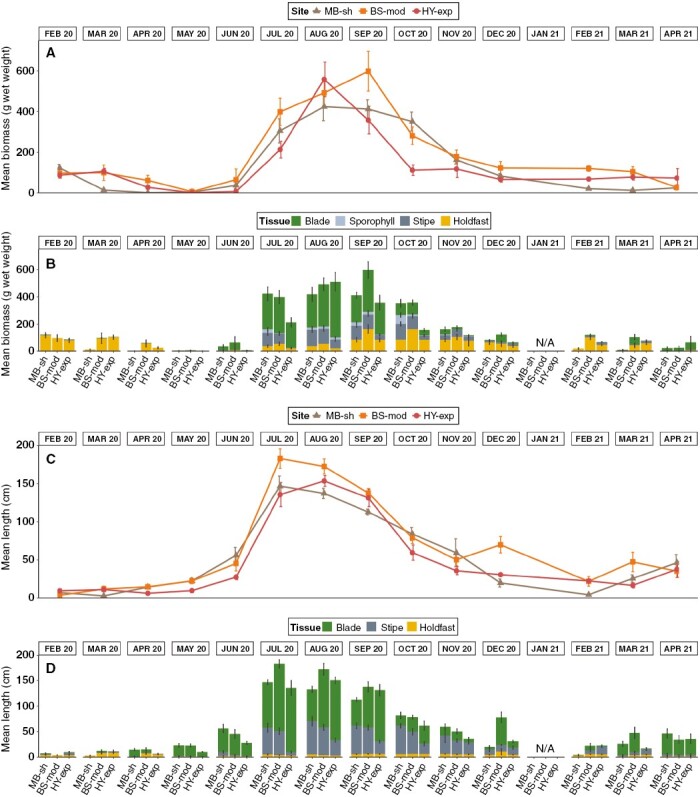
Spatiotemporal variability in biometric measurements from individuals sampled from intertidal *S. polyschides* populations. Mean total biomass (A, B) and total length (C, D) (± s.e.). Stacked bar plots indicate values for each structural tissue component.

At all intertidal sites, sporophyte size and biomass exhibited pronounced seasonality, increasing throughout the development period from a juvenile recruit to full maturity, followed by a decrease in biomass and size during the period of decay and senescence (Fig. 4). Maximum mean biomass (Fig. 4A, B) of 598.1 ± 97.8 g was recorded in September at BS-mod, whereas maximum mean length (Fig. 4C, D) of 182.95 ± 12.8 cm was recorded in July 2020 at BS-mod. During periods of peak biomass (i.e. summer months), the structural blade compartment consisted of ~50 % of the total sporophyte weight, whereas the relative contribution of holdfasts to total weight was greater in other periods (Fig. 4B). With regard to total length, the relative contribution of blade structures was generally greater than that of stipes and holdfasts (Fig. 4D).

**Fig. 5.**
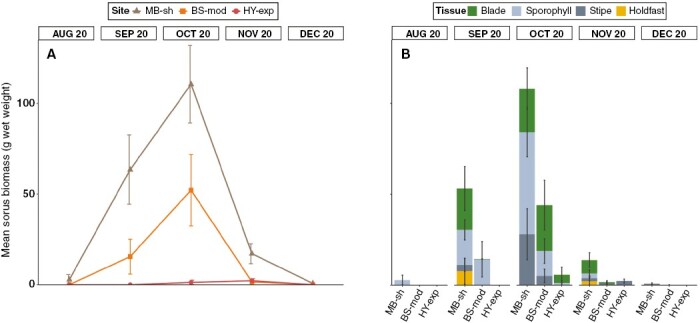
Spatiotemporal variability in sorus biomass from intertidal *S. polyschides* populations. (A) Mean biomass (± s.e.) of fertile sorus tissue (wet weight) and (B) mean biomass of sorus associated with each structural component during main reproductive months.

Development of mature sorus was recorded between August and December 2020 (Fig. 5). Additionally, minor sorus tissue production outside the fertile summer months was recorded in March and April 2021 in some individuals. We recorded clear differences between sites in the weight of sorus tissue (Fig. 5A), with the highest values at the least exposed/most sheltered site (MB-sh) and lowest sorus production at the most exposed site (HY-exp). Sorus tissue was recorded on all structural compartments of the sporophyte (Fig. 5B), not only on the sporophyll. The most prominent fertile sporophyll tissue and highest sporophyll biomass were observed in September and October (Fig. 5B). Univariate PERMANOVA detected significant differences in all response variables between the main factors of ‘site’ and ‘month’, as well as the interaction term (Table 1). Post-hoc tests within the interaction term of main factors (Supplementary Data S3) showed that the magnitude of differences between sites varied across months, but did not exhibit a clear seasonal pattern.

**Fig. 6.**
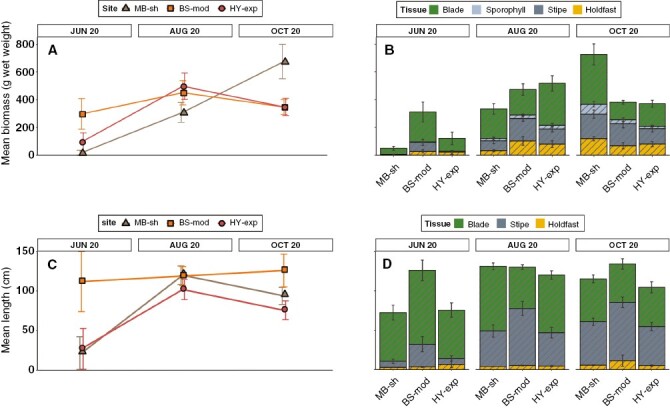
Spatiotemporal variability in biometric measurements from individuals sampled from subtidal *S. polyschides* populations. Mean total biomass (A, B) and total length (C, D) (± s.e.). Stacked bar plots indicate values for each structural tissue component. Note that subtidal survey months are not consecutive.

For subtidal populations, sporophyte biomass and length increased from June to August at all sites (to varying degrees), while trends from August to October varied between sites (Fig. 6). Maximum mean sporophyte biomass was observed at MB-sh in October (655.3 ± 114.4 g), with blades comprising ~50 % of total biomass (Fig. 6A, B). Maximum mean length was recorded at BS-mod in October (134.3 ± 12.3 cm) and was more consistent between sites and sampling events (Fig. 6C, D). Univariate PERMANOVA detected a significant ‘site’ × ‘month’ interaction term for biomass (Table 2) with post-hoc tests indicating that sites did not differ in August and October but did in June (Supplementary Data S4). A significant ‘site × month’ interaction term for sorus biomass, however, indicated differences in August and October, but not in June. Total sporophyte length varied between the main factors of site (pairwise post-hoc test: BS-mod > HY-exp = MB-sh) and month (pairwise post-hoc test: June < August = October). In general, sporophyte biomass and length in subtidal habitats was greater than that observed in intertidal habitats for the corresponding months of June and October, but less in the intermediate month (August).

### Standing stock

**Fig. 7.**
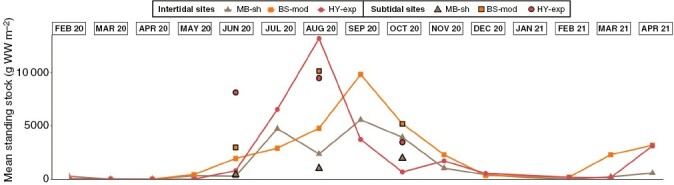
Spatiotemporal variability of estimated mean standing stock biomass for *S. polyschides* populations in both intertidal (lines and symbols) and subtidal (bold symbols only) habitats.

Estimates of standing stock ranged from 0 to over 13 000 g WW m^−2^ and exhibited high variability between sites and months (Fig. 7). For intertidal populations, standing stock peaked at HY-exp in August (13 176 g WW m^−2^), and at BS-mod (9809 g WW m^−2^) and MB-sh (5565 g WW m^−2^) in September, following rapid increases from June onwards (Fig. 7). Standing stock declined rapidly after September, returning to negligible values through winter when holdfasts represented the majority of the biomass. Univariate PERMANOVA detected a significant interaction term (Table 1), with post-hoc tests indicating that differences between sites did not persist in all months (Supplementary Data S3).

For subtidal populations, estimates of standing stock were greatest at BS-mod (10 132 g WW m^−2^) and HY-exp (9476 g WW m^−2^) in August, and peaked in October at MB-sh (1966 g WW m^−2^) (Fig. 7). On average, standing stock at HY-exp (more exposed site) was about six times greater than at MB-sh (more sheltered site). Indeed, univariate PERMANOVA detected a significant main effect of ‘site’ (Table 2), with higher standing stock values recorded at HY-exp and BS-mod compared with least exposed MB-sh. In general, standing stock estimates were lower in the sheltered/moderate sites than at the more exposed sites in the intertidal as well as in the subtidal zone (MB-sh < BS-mod < HY-exp). In June, standing stock estimates for subtidal populations were four-fold higher than those of intertidal populations, but estimates were more comparable between habitats by August and October.

Due to the pseudo-annual growth strategy of *S. polyschides*, maximum standing stock values can be considered proxies for annual biomass accumulation, as almost all organic matter fixed later senesces and is released into the environment. As such, estimates of biomass accumulation across both depths (mean of intertidal and subtidal measurements) were lowest at the most wave-sheltered site (3765 g WW m^−2^ year^−1^ at MB-sh) and markedly higher at the more wave-exposed sites (9970 g WW m^−2^ year^−1^ at BS-mod; 11 326 g WW m^−2^ yr^−1^ at HY-exp).

## DISCUSSION

We present the first study on population demography of *S. polyschides* situated within the central area of the species’ range, in the Western English Channel (southwest UK). Previous research on the population dynamics of *S. polyschides* and their associated communities in the UK was conducted 40–60 years ago (Burrows, 1958; Norton, 1968, 1969, 1971, 1977; Norton and Burrows, 1969), towards the (former) leading range edge on the Isle of Man (54°N).

In our study, we observed marked seasonality in the density, coverage, biomass, morphology and standing stock of *S. polyschides*, with relatively minimal variation between sites situated along a gradient of wave exposure, and depths. Density and cover data show an expected structural development of individuals and an ‘ageing’ population through several sporophyte life stages during one growth season: progressing from juvenile to healthy adult sporophytes in spring and summer, followed by a senescing population in late autumn and winter.

Along the shores of the Western English Channel, *S. polyschides* usually forms mixed stands with ‘true’ Laminarian kelps. Intertidal stands are typically characterized by *L. digitata* and to a lesser extent *Saccharina latissima* and *U. pinnatifida* (Yesson *et al.*, 2015*a*; Epstein *et al.*, 2019), while subtidal habitats are typically dominated by *L. hyperborea* and, to a lesser extent, *L. ochroleuca* (Smale and Moore, 2017; Smale *et al.*, 2022). Our study showed that these mixed kelp stands are, in fact, dominated by *S. polyschides* in summer, which provides substantial habitat and covers over 50 % of the rocky substrate. Our estimates of the standing stock of *S. polyschides* peaked at ~13 000 g WW m^−2^ in late summer. We recorded peak production of fertile sorus tissue in late summer/early autumn. In winter, no fertile tissue was recorded, and population density and standing stock were drastically reduced. Only a small component of the population remained as remnant holdfasts throughout winter, persisting until the beginning of the following recruitment season. The marked contrast from dense space-occupying *S. polyschides* populations in summer to collapsed, remnant populations in winter is driven by the pseudo-annual life cycle of the species as described by Norton and Burrows (1969).

We recorded some variation in population dynamics across our survey sites, which were situated along a gradient of wave exposure. It is well established that variation in wave exposure can influence kelp population demography, by affecting settlement, recruitment, sporophyte density and morphology, productivity, and fitness (Smale *et al.*, 2011; Burrows, 2012; Pedersen *et al.*, 2012). For example, positive responses to increasing wave exposure have been reported for *Sargassum muticum* in Ireland (Baer and Stengel, 2010), whereas negative responses to increasing wave exposure have been recorded for both the non-native kelp *U. pinnatifida* in SW England (Epstein and Smale, 2017*a*) and the giant kelp *Macrocystis pyrifera* in California (Graham *et al.*, 1997). As such, site-level variability between populations of *S. polyschides* may have been driven, at least in part, by differences in wave exposure, although other abiotic and biotic factors can drive variability at this spatial scale, including competition (Epstein *et al.*, 2019), grazing pressure (Plouguerné *et al.*, 2006), habitat topography (Harries *et al.*, 2007*a*, *b*) and oceanographic features (e.g. upwelling, freshwater input, turbidity) (Fernandez *et al.*, 1988; Fernández, 2011; Pereira *et al.*, 2015; Bermejo *et al.*, 2016).

We recorded greater production of sorus material under sheltered rather than exposed conditions (MB-sh > BS-mod > HY-exp), whereas peak standing stock, and therefore annual productivity, was greater under exposed rather than sheltered conditions (HY-exp > BS-mod > MB-sh), in both intertidal and subtidal habitats. This suggests that wave exposure could influence growth and energy strategies, as has been shown for other kelp species (Buschmann *et al.*, 2006; Pedersen *et al.*, 2012). However, the causative effect of wave exposure on the production of fertile material by *S. polyschides* sporophytes remains speculative at this point.

We recorded a high density of late-season (October) recruits in subtidal populations, similar to observations by Norton and Burrows (1969), who recorded recruitment and the presence of mature sporophytes in subtidal populations throughout the whole year at the Isle of Man (~54°N). Reduced wave action and lower thermal stress in subtidal, compared with intertidal, habitats might favour year-round development of recruits, increase sporophyte longevity and lead to greater standing stock in the late season. For example, at the end of the peak-growth season (October), we recorded greater biomass and total length of sporophytes in subtidal compared with intertidal habitats, where sporophytes showed increased shredding of blade tissue and signs of decay. That said, the maximum recorded standing stock of *S. polyschides* did not differ significantly between depths.

*Saccorhiza polyschides* sporophytes were shorter-lived than perennial *Laminaria* species, and, as such, offered less stable and persistent habitat for associated species, such as epiphytes, benthic invertebrates but also small fishes (Salland and Smale, 2021). Similarly, standing stock within *S. polyschides* populations was strongly seasonal, with maximum values in late summer/autumn, followed by a period of release of organic matter into the environment as detritus. This intense late-season pulse of detritus release is in contrast to dominant laminarial kelp species in the northeast Atlantic, which either release a pulse of detritus in spring or release detritus more gradually through the year (Pessarrodona *et al.*, 2018*a*; Gilson *et al.*, 2023). Differences in biomass accumulation and detritus production between dominant species may have implications for local carbon cycling or supply for detrital foodwebs (Gilson *et al.*, 2021; Guerrero-Meseguer *et al.*, 2023). The mean standing stock of *S. polyschides*, across all sites and surveys, was ~2300 g WW m^−2^, which is about one-third of the average standing stock values reported for the dominant kelp *L. hyperborea* in the southwest of the UK (Smale *et al.*, 2016, 2020). However, due to the pronounced seasonality in the productivity of *S. polyschides*, maximum observed standing stock values exceeded 13 000 g WW m^−2^. This is more than double the annual mean standing stock values reported for *L. hyperborea* populations in the UK.

*Saccorhiza polyschides* is an opportunistic kelp that is thought to be thriving and proliferating along the Western English Channel (Birchenough and Bremner, 2010; Smale *et al.*, 2013, 2015). To date, several studies on *S. polyschides* have been conducted towards its southern distribution range (Iberia–Morocco), where this species can be (or has been) locally dominant and a key habitat-former (Fernández, 2011; Díez *et al.*, 2012; Tuya *et al.*, 2012; Voerman *et al.*, 2013; Pereira, 2014; Assis *et al.*, 2017; Casado-Amezúa *et al.*, 2019). Along the coasts of Morocco and the Iberian Peninsula, population declines have been reported (Fernández, 2011; Díez *et al.*, 2012; Assis *et al.*, 2017; Casado-Amezúa *et al.*, 2019), probably as a result of ocean warming leading to unfavourable thermal conditions, as well as shifts in nutrient availability related to upwelling events (Lüning, 1985; Fernandez *et al.*, 1988; Fernández, 2011; Pereira *et al.*, 2015). In a comparable study on seasonal dynamics of intertidal *S. polyschides* populations, Pereira *et al.* (2015) found contrasting patterns between populations from different latitudes. Populations persisting at lower latitudes (~41°N) exhibited shorter seasonal life cycles with sporophytes only present between April and September, whereas populations found at higher latitudes (~48°N) exhibited year-round presence and recruitment of sporophytes. In the current study (~50°N), we also observed the presence of sporophytes throughout the year but recruitment of sporophytes into intertidal populations exhibited seasonality, with the highest rates in spring and summer. Clearly, population dynamics differ across the distribution of *S. polyschides*, probably due to variation in environmental conditions and/or species interactions, and further research on patterns of spatiotemporal variability of *S. polyschides* populations across its wider biogeographical range in the northeast Atlantic is warranted.

## CONCLUSION

Our study showed that *S. polyschides* is a conspicuous and seasonally dominant component of macroalgal assemblages on rocky reefs towards the centre of its range (i.e. the Western English Channel), where it probably contributes considerably to ecosystem functioning through habitat provisioning and biomass accumulation and release. We observed distinct seasonal variability, characterized by high recruitment in spring, peak sporophyte biomass and high standing stock in summer, followed by declines in density, cover and biometric measurements during winter. These patterns exhibited a reasonable level of consistency across sites and depths. By late summer, *S. polyschides* was a dominant habitat-former with maximum mean standing stock > 13 000 g WW m^−2^, providing a complex and abundant biogenic habitat for associated organisms.

*Saccorhiza polyschides* is a warm-tolerant, opportunistic species with high local population densities. It has probably proliferated in recent years across the southwest of the UK (Smale *et al.*, 2013), and has extended, or is predicted to extend, its distribution polewards (Yesson *et al.*, 2015*b*; Assis *et al.*, 2017, 2020). As such, we expect this species to become increasingly dominant under projected climate change, particularly in response to ocean warming, increased storminess and other environmental changes. Our study provides a robust baseline on the population demographics of *S. polyschides* against which to detect future changes in the Western English Channel, although more information on interannual variability and long-term trends is needed. More generally, it offers a useful case study and approach and provides a foundation for further research on the ecological role of the species within the wider temperate reef ecosystem.

## SUPPLEMENTARY DATA

Supplementary data are available online at https://academic.oup.com/aob and consist of the following. SI2: Detailed method description/age classification of *S. polyschides*. SI3: Post-hoc table. SI4: Post-hoc table.

mcad181_suppl_Supplementary_Material
