# Supplementary material for: Spatiotemporal variability in population demography and morphology of the habitat-forming macroalga Saccorhiza polyschides in the Western English Channel
Source: Ann Bot. 2023 Nov 14;133(1):117–30. doi: 10.1093/aob/mcad181 (PMC10921834; doi:10.1093/aob/mcad181)
Supplement: mcad181_suppl_Supplementary_Material [file mcad181_suppl_supplementary_material.docx]

Supplementary Material

The following supplement accompanies the ‘Research in context’ article in ‘Annals of Botany’

**Spatiotemporal variability in population demography and morphology of the habitat-forming macroalga *Saccorhiza polyschides* in the Western English Channel**

Nora Salland^*^, Catherine Wilding, Antony Jensen, Dan A. Smale ^* norsal@mba.ac.uk^

**Supplementary Information S1:** Detailed method description / Survey site details

Geographical and environmental characteristics for each survey site.

| site | | **MB-sh** | **BS-mod** | **HY-exp** |
| --- | --- | --- | --- | --- |
| local name | | Mount Batten (**MB**) | Bovisand (**BS**) | Heybrook (**HY**) |
| intertidal | latitude (°N)  longitude (°W) | 50°21’32.7’’  04°07’52.8’’ | 50°19’55.4’’  04°07’13.8’’ | 50°19’08.4’  04°06’49.6’’ |
| subtidal | latitude (°N)  longitude (°W) | 50°21’27.1’’  04°07’53.4’’ | 50°19’34.3’’  04°07’34.8’’ | 50°19’02.5’  04°06’57.6’’ |
| log wave fetch (km) ***** | | 2.911 | 3.735 | 3.918 |
| wave exposure | | sheltered (**sh**) | moderate (**mod**) | exposed (**exp**) |
| reef type and locality | | deepening slopes with large, silty gullies | flat platforms with gullies surrounded by sandy beach | deepening slopes and gullies; some flat platforms |

*‘Log wave fetch’ is a broad-scale metric of wave exposure, It is calculated by summing the fetch values computed for 32 angular sectors around each study site (see Burrows, 2012).

**Supplementary Information S2:** Detailed method description / Age classification of *S. polyschides:*

In this field-based survey, microscopic life stages of *S. polyschides* (spores, gametophytes, microscopic sporophytes) were not considered. Similarly to Norton and Burrows (1969), we differentiated the life cycle of the sporophyte into three ages classes. However, our classification (see Fig. 1) is mainly based on the development of the bulb and total sporophyte length.

1. Juveniles (Fig. 1C): Recruit clearly identifiable by its dark spot on the stipe ("small circular ridge"; see Norton and Burrows, 1969), later developing into the upper part of the holdfast (see Fig. 1C, left). Developing bulb reminding of an “umbrella” (Barber, 1889), “bell” (Svendsen, 1962, Norton and Burrows, 1969), or mushroom, not enclosed and connected with the lower haptera and substratum (see Fig. 1D, right). Stipe first round later flat, but not yet twisted. Blade usually single, exceptionally several blade digits, ranging from about 3.5 cm to 35 cm (with some larger exemptions, especially in the subtidal samples).
2. Healthy, mature adults (Fig 1D): Bulbous holdfast complete developed and upper holdfast connected with lower haptera and substratum. Thallus complete (total length usually 35-250 cm, depending on the season). Stipe is flat, twisted at base (above holdfast), later with ‘frills’ (fruity tissue; when mature, dark patches of spore-filled sorus tissue appear), without signs of decay. Blade with several digits (ranging between three to > 30). Tissue loss on blade common by late summer due to shredding.
3. Senescing/decaying/deceasing, ‘old’ adults (Fig 1E): Blade and/or stipe partly or completely decayed. Sometimes only holdfast tissue remains attached to rocky substrate, but clearly identifiable as *S. polyschides* by its holdfast and plump haptera. See also overwintering senescing holdfast characteristics and morphology in Salland and Smale (2021).

**Supplementary Information S3:** Post-hoc table

Post-hoc results for interaction terms of univariate PERMANOVA ‘site x month’ in the intertidal. Total sporophyte density (A), total sporophyte cover (B), total biomass (C), total length (D), sorus biomass (E), and standing stock (F) (see Table 1). Colouration of field indicate the significance of interaction: ■ high interaction (*p* ≤ 0.001), ■ medium interaction (*p* ≤ 0.01), ■ low interaction (*p* ≤ 0.05), ■ no interaction (*p* > 0.05).

| **A. density** | **FEB 20** | **MAR 20** | **APR 20** | **MAY 20** | **JUN 20** | **JUL 20** | **AUG 20** | **SEP 20** | **OCT 20** | **NOV 20** | **DEC 20** | **JAN 21** | **FEB 21** | **MAR 21** | **APR 21** |
| --- | --- | --- | --- | --- | --- | --- | --- | --- | --- | --- | --- | --- | --- | --- | --- |
| MB-sh ~ BS-mod |  |  |  |  |  |  |  |  |  |  |  | N/A |  |  |  |
| MB-sh ~ HY-exp |  |  |  |  |  |  |  |  |  |  |  | N/A |  |  |  |
| BS-mod ~ HY-exp |  |  |  |  |  |  |  |  |  |  |  | N/A |  |  |  |
| **B. cover** | **FEB 20** | **MAR 20** | **APR 20** | **MAY 20** | **JUN 20** | **JUL 20** | **AUG 20** | **SEP 20** | **OCT 20** | **NOV 20** | **DEC 20** | **JAN 21** | **FEB 21** | **MAR 21** | **APR 21** |
| MB-sh ~ BS-mod | N/A | N/A | N/A |  |  |  |  |  |  |  |  | N/A |  |  |  |
| MB-sh ~ HY-exp | N/A | N/A | N/A |  |  |  |  |  |  |  |  | N/A |  |  |  |
| BS-mod ~ HY-exp | N/A | N/A | N/A |  |  |  |  |  |  |  |  | N/A |  |  |  |
| **C. total biomass** | **FEB 20** | **MAR 20** | **APR 20** | **MAY 20** | **JUN 20** | **JUL 20** | **AUG 20** | **SEP 20** | **OCT 20** | **NOV 20** | **DEC 20** | **JAN 21** | **FEB 21** | **MAR 21** | **APR 21** |
| MB-sh ~ BS-mod |  |  |  |  |  |  |  |  |  |  |  | N/A |  |  |  |
| MB-sh ~ HY-exp |  |  |  |  |  |  |  |  |  |  |  | N/A |  |  |  |
| BS-mod ~ HY-exp |  |  |  |  |  |  |  |  |  |  |  | N/A |  |  |  |
| **D. total length** | **FEB 20** | **MAR 20** | **APR 20** | **MAY 20** | **JUN 20** | **JUL 20** | **AUG 20** | **SEP 20** | **OCT 20** | **NOV 20** | **DEC 20** | **JAN 21** | **FEB 21** | **MAR 21** | **APR 21** |
| MB-sh ~ BS-mod |  |  |  |  |  |  |  |  |  |  |  | N/A |  |  |  |
| MB-sh ~ HY-exp |  |  |  |  |  |  |  |  |  |  |  | N/A |  |  |  |
| BS-mod ~ HY-exp |  |  |  |  |  |  |  |  |  |  |  |  |  |  |  |
| **E. sorus biomass** | **FEB 20** | **MAR 20** | **APR 20** | **MAY 20** | **JUN 20** | **JUL 20** | **AUG 20** | **SEP 20** | **OCT 20** | **NOV 20** | **DEC 20** | **JAN 21** | **FEB 21** | **MAR 21** | **APR 21** |
| MB-sh ~ BS-mod |  |  |  |  |  |  |  |  |  |  |  | N/A |  |  |  |
| MB-sh ~ HY-exp |  |  |  |  |  |  |  |  |  |  |  | N/A |  |  |  |
| BS-mod ~ HY-exp |  |  |  |  |  |  |  |  |  |  |  | N/A |  |  |  |
| **F. standing stock** | **FEB 20** | **MAR 20** | **APR 20** | **MAY 20** | **JUN 20** | **JUL 20** | **AUG 20** | **SEP 20** | **OCT 20** | **NOV 20** | **DEC 20** | **JAN 21** | **FEB 21** | **MAR 21** | **APR 21** |
| MB-sh ~ BS-mod |  |  |  |  |  |  |  |  |  |  |  | N/A |  |  |  |
| MB-sh ~ HY-exp |  |  |  |  |  |  |  |  |  |  |  | N/A |  |  |  |
| BS-mod ~ HY-exp |  |  |  |  |  |  |  |  |  |  |  | N/A |  |  |  |

**Supplementary Information S4:** Post-hoc table

Post-hoc results for significant interaction terms of univariate PERMANOVA ‘site x month’ in the subtidal. Total sporophyte density (A), total sporophyte cover (B), total biomass (C), sorus biomass (D) (see Table 2). Colouration of field indicate the significance of interaction: ■ high interaction (*p* ≤ 0.001), ■ medium interaction (*p* ≤ 0.01), ■ low interaction (*p* ≤ 0.05), ■ no interaction (*p* > 0.05). Note that months are not consecutive here.

| **A. density** | **JUN 20** | **AUG 20** | **OCT 20** |
| --- | --- | --- | --- |
| MB-sh ~ BS-mod |  |  |  |
| MB-sh ~ HY-exp |  |  |  |
| BS-mod ~ HY-exp |  |  |  |
| **B. cover** | **JUN 20** | **AUG 20** | **OCT 20** |
| MB-sh ~ BS-mod |  |  |  |
| MB-sh ~ HY-exp |  |  |  |
| BS-mod ~ HY-exp |  |  |  |
| **C. total biomass** | **JUN 20** | **AUG 20** | **OCT 20** |
| MB-sh ~ BS-mod |  |  |  |
| MB-sh ~ HY-exp |  |  |  |
| BS-mod ~ HY-exp |  |  |  |
| **D. sorus biomass** | **JUN 20** | **AUG 20** | **OCT 20** |
| MB-sh ~ BS-mod |  |  |  |
| MB-sh ~ HY-exp |  |  |  |
| BS-mod ~ HY-exp |  |  |  |

**References: Supplementary Information**

BARBER, C. A. 1889. On the structure and development of the bulb in *Laminaria bulbosa*, Lamour. *Annals of Botany,* 3**,** 41-64.

BURROWS, M. T. 2012. Influences of wave fetch, tidal flow and ocean colour on subtidal rocky communities. *Marine Ecology Progress Series,* 445**,** 193-207. doi: 10.3354/meps09422.

NORTON, T. A. & BURROWS, E. M. 1969. Studies on marine algae of the British Isles. 7. *Saccorhiza polyschides* (Lightf.) Batt. *British Phycological Journal,* 4**,** 19-53. doi: 10.1080/00071616900650031.

SALLAND, N. & SMALE, D. 2021. Spatial variation in the structure of overwintering, remnant *Saccorhiza polyschides* sporophytes and their associated assemblages. *Journal of the Marine Biological Association of the United Kingdom,* 101**,** 639-648. doi: 10.1017/S0025315421000692.

SVENDSEN, P. 1962. Some observations on *Saccorhiza polyschides* (Lightf.) batt. (Phaeophyceae). *Sarsia,* 7**,** 11-13. doi: 10.1080/00364827.1962.10410263.
